# The Hippo Transducer YAP/TAZ as a Biomarker of Therapeutic Response and Prognosis in Trastuzumab-Based Neoadjuvant Therapy Treated HER2-Positive Breast Cancer Patients

**DOI:** 10.3389/fphar.2020.537265

**Published:** 2020-08-27

**Authors:** Jia-qi Yuan, Nian-hua Ding, Zhi Xiao

**Affiliations:** ^1^ Clinical Research Center For Breast Cancer Control and Prevention in Hunan Province, Department of General Surgery, Xiangya Hospital, Central South University, Changsha, China; ^2^ Department of Clinical Laboratory, The First Hospital of Changsha, Changsha, China

**Keywords:** Trastuzumab-based neoadjuvant therapy, YAP/TAZ, HER2/HER3 heterodimer, breast cancer-free interval, SKBR-3 cell lines

## Abstract

**Background:**

We explored the therapeutic and prognostic effect of YAP/TAZ intensityinHER2-positive breast cancer patients. We also investigated the relationship between YAP/TAZ expression and Trastuzumab-resistance.

**Methods:**

We collected clinicopathological information from 397 cases. We evaluated therapeutic and prognostic effect of YAP/TAZ and other variables. We also cultivated Trastuzumab-resistance cell lines and explored relationship between YAP/TAZ and Trastuzumab-resistance.

**Results:**

Over-expression of YAP/TAZ was remarkable in Trastuzumab-resistant cells, and so did HER3 and HER2/HER3 heterodimer. Inhibition of YAP/TAZ expression reversed Trastuzumab-resistance.YAP/TAZ deficiency contributed to favorable therapeutic response, and so did hormone receptor insufficiency and chemotherapy dosage inferiority. Deficient YAP/TAZ intensity and abundant hormone receptor intensity contributed to better survival. Over-expression of YAP/TAZ was obvious in recurrent cases in comparison with their matching primary lesions. Prognostic superiority of insufficient YAP/TAZ intensity was more outstanding in hormone receptor negative cases. Over-expression of YAP/TAZ and HER3 was generally synchronous. Absence of HER3 expression in residual lesions might correlate with better breast cancer-free survival.

**Conclusions:**

Over-expression of YAP/TAZ as well as HER-3 and HER2/HER3 heterodimer was synchronously remarkable in Trastuzumab-resistant cell lines. Inhibition of YAP/TAZ expression reversed Trastuzumab resistance. Deficient YAP/TAZ intensity as well as insufficient hormone receptor intensity and high chemotherapy dosage contributed to favorable therapeutic response. Deficient YAP/TAZ intensity and abundant hormone receptor intensity contributed to better survival, and so did absence of HER3expression in residual lesions. Prognostic superiority of YAP/TAZ expression depended on hormone receptor status. Cases with synchronous over-expression of YAP/TAZ and HER3 suffered poor survival, which revealed the potential effect of YAP/TAZ-HER2/HER3 crosstalk in prognosis of HER2-positive patients.

## Introduction

Gene amplification resulted in over-expression of the human epidermal growth factor receptor 2 (HER2), which induced shorter disease-free survival and decreased overall survival. Fortunately, routine use of Trastuzumab altered the natural history of HER2 positive breast cancer (Mittendorf et al., 2009). Trastuzumab was a monoclonal antibody that targeted the extracellular domain of HER2 protein and interfered with HER2-mediated signaling cascade, preventing proliferation and eventually leading to cell death (Hudis, 2007). Adjuvant use of Trastuzumab reduced relapse in HER2 positive cases (Perez et al., 2011). Neoadjuvant use of Trastuzumab improved pathological complete response rates (Gianni et al., 2010).

Despite these successes, we noticed that some cases did not respond well to Trastuzumab. For HER2 positive breast cancer patients, disease relapse might occur even after standard anti-HER2 therapy (He et al., 2011; Zhou et al., 2014; Yuan et al., 2015; Zhu et al., 2017; Zhu et al., 2018). Previous literatures indicated that Hippo pathway was an evolutionarily conserved regulator for tissue development (Harvey et al., 2013). Mutations of pathway components caused uncontrolled tissue overgrowth (Tapon et al., 2002), revealing some kind of tumorigenicity (Camargo et al., 2007). Crosstalk of Hippo signaling with other perturbed molecular networks might result in the happening of tumor invasion (Johnson and Halder, 2014). The central role of Hippo pathway focused on degrading of two homologous oncoproteins: the transcriptional co-activator with PDZ-binding motif (TAZ) and Yes-associated protein (YAP). Preliminary clinical studies from a consecutive series of breast cancer patients found that YAP/TAZ over-expression related to shorter disease-free survival, and a statistically obvious correlation between YAP/TAZ and HER2 positivity had also been proved (Bartucci et al., 2015).

In this study, we explored the therapeutic and prognostic effect of YAP/TAZ expression. We also investigated relationship between YAP/TAZ expression and Trastuzumab resistance. We hypothesized that YAP/TAZ-HER2/HER3 crosstalk affected the prognosis of HER2-positive cases. We estimated prognostic effect of YAP/TAZ and HER2/HER3 heterodimer according to our preclinical and clinical findings.

## Materials and Methods

### Study Population

This study enrolled 397 pathology confirmed HER2 positive breast cancer patients from the Breast Cancer Center, Xiangya Hospital, Central South University, between 2012.3 and 2018.3. We excluded patients who suffered inflammatory breast cancer, distant metastasis disease, or bilateral breast tumors. The median follow-up time was 48 months (22–69 months). Xiangya Hospital Ethics Committee reviewed and approved all involved cases. The patients provided their written informed consent to engage in this study.

### Study Design and Procedures

In this retrospective study, we gained pathological diagnose *via* core needle biopsy. Cytotoxic therapy was anthacycline and taxane intravenously every 21 days for 8 cycles. Trastuzumab treatment was 8 mg/kg as a loading dose, and then 6mg/kg every 3 weeks for 1 year. All involved cases underwent the above neoadjuvant therapy (NAT). All patients received proper surgical procedure (breast-conserving surgery or modified radical mastectomy) within 1 month after NAT finished. Considering the false negative results of sentinel lymph nodes biopsy after NAT, all patients underwent axillary lymph nodes dissection. Local advanced cases and breast-conserving cases received radiation therapy. Hormone receptor (HR) positive cases underwent proper adjuvant endocrine therapy (**Table 1**).

**Table 1:** Demographic information of subjects in this study.

Characteristic	No. (n=397)	%
Age (years)		
Median (range)	45 (21-74)	–
≤50	254	63.98
>50	143	36.02
cTNM stage		
II	191	48.11
III	206	51.89
Menstrual status		
Menopause	188	47.36
Non menopause	209	52.64
Histological grade		
I-II	216	54.41
III	181	45.59
Ki67 score (%)		
>14%	223	56.17
≤14%	174	43.83
HR status		
HR+	240	60.45
HR-	157	39.55
YAP/TAZ score		
≤0.5	226	56.93
>0.5	171	43.07
Local therapy^1^		
Mastectomy+ALND+RT	87	21.91
Mastectomy+ALND	236	59.45
BCS+ALND+RT	74	18.64
Pathological response		
pCR	104	26.20
Non-pCR	293	73.80
Lymph nodes after NAC		
>3	281	70.78
≤3	116	29.22
RTDI		
>85%	273	68.77
≤85%	124	31.23

^1^ALND, axillary lymph node dissection; BCS, breast conserving surgery; RT, radiation therapy.

Formalin-fixed and paraffin-embedded specimens were finally manufactured into 4μm-thick slices and then stained by Hematoxylin and eosin (H&E). All pathological data was available, such as estrogen receptor (ER), progesterone receptor (PR), HER2, and Ki-67 indication. We evaluated signals in both core needle biopsy specimens and residual tumors. The positive status of HR was immunohistochemistry (IHC) staining ER≥1% and/or PR≥1%.The positive status ofHER2 was IHC staining HER2 3+ or FISH +.The positive status of FISH was single-probe average HER2 copy number ≥6.0 signals/cell; or dual-probe HER2/CEP17 ratio of ≥2.0with an average HER2 copy number ≥4.0 signals/cell; or dual-probe HER2/CEP17 ratio of <2.0 with an average HER2 copy number ≥6.0 signals/cell (Wolff et al., 2018).

Our group employed anti-YAP/TAZ to evaluate YAP/TAZ status in diagnostic biopsy specimens (primary lesions and recurrent lesions). We confirmed the positive status of YAP/TAZ when more than 10% tumor cells were nuclear and/or cytoplasmic staining. We graded the IHC staining intensity of YAP/TAZ as 0 (negative), 1+ (weak), 2+ (moderate), and 3+ (strong). The calculation method of YAP/TAZ score was multiplying IHC intensity by 1.5 in nuclear stained cases and by0.5 in cytoplasmic stained cases (summing them together in both nuclear and cytoplasmic stained cases). Median score ≤0.5 was YAP/TAZ low expression, whereas median score >0.5 was YAP/TAZ high expression. Two investigators assessed the pathological data independently (Vici et al., 2014).

This study defined pathological complete response (pCR) as no residual invasive breast or lymph node lesions after NAT. We employed ultrasound and MRI every 21 days to assess the residual tumor size. We evaluated the clinical response according to the criteria described in solid tumor (RECIST) guideline version 1.1. We calculated objective response rate (ORR) by comparing complete response (CR) and partial response (PR) cases to the total number of involved cases.

### Calculation of Dose Intensity

Relative total dose intensity (RTDI): ratio of actual total dose intensity (ATDI) and planned total dose intensity (PTDI) (Loibl et al., 2011).

$$\text{RTDI}(\%)=\frac{\text{ATDi}}{\text{PTDI}}\times 100$$

Planned total dose intensity (PTDI): the planned total dose and the planned treatment duration, average across the chemotherapy agents used.

$$\text{PTDI}(\text{mg}/\text{week})=\frac{\text{Planned}\;\text{Total}\;\text{Dose}\;(\text{mg})}{\text{planned}\;\text{duration}\;\text{of}\;\text{therapy}\;(\text{weeks})}$$

Actual total dose intensity (ATDI): the ratio of actual total dose and the real treatment duration.

$$\text{ATDI}(\text{mg}/\text{week})=\frac{\mathrm{Actual}\;\text{Total}\;\text{Dose}\;(\text{mg})}{\text{duration}\;\text{of}\;\text{therapy}\;(\text{weeks})}$$

After calculated separately for each component of the regimen, an average was taken to obtain the final RTDI of the combination.

$$\text{RTD}{\text{I}}_{\text{TAC}}=\frac{\text{RTD}{\text{I}}_{\text{T}}+\text{RTD}{\text{I}}_{\text{A}}+\text{RTD}{\text{I}}_{\text{C}}}{3}$$

In this study, we employed SKBR-3 cell line to cultivate Trastuzumab-resistance cell model. We calculated the growth rate of cells *via* colorimetric method. We employed YAP/TAZ inhibitor-1 (Medchemexpress LLC, New Jersy, USA) to evaluate the relationship of YAP/TAZ expression and Trastuzumab resistance. We assessed YAP/TAZ expression according to their localization by western blot analysis. We also used immunodetection assay to estimate the expression of HER3 and HER2/HER3 heterodimer.

### Statistical Analysis

We used one-way analysis of variance to clarify relationship between variables and clinical response. We employed logistical regression to explore the impact of variables on pathological remission rate. Univariate analysis and Pearson χ2 test were both qualified to evaluate the effect of relevant variables on clinical and pathological response in different subgroups. Breast cancer-free interval (BCFI) was proper for the survival analyses, which was the time between surgery and first invasive relapse (local or distant).Cox proportional hazards model was useful to evaluate the prognostic effect of variables, and the results were expressed as hazard ratios (HRs) and 95% confidence intervals (CIs). We employed Kaplan-Meier survival analysis to estimate the relationship between variables and prognosis. We also performed sub-population treatment effect pattern plot (STEPP) methodology and standard method for competing risk analysis to evaluate the disease-specific cumulative incidence and composite recurrence risk. All statistical tests were two-sides and *p* values less than 0.05 were considered statistically significant. We carried out STEPP analysis using the R software package (R Foundation for Statistical Computing, Vienna, Austria; https://sites.google.com/site/stepprpackage). We carried out other statistical analysis by SPSS version 19.0 for Windows (SPSS Inc, Chicago, USA). Xiangya Clinical Institutional Review Board approved this study. We obtained approvals from the institutional review board before the study procedures began.

## Results

### Cell Culture and Pharmacological Treatment

Our group noted down cell proliferation at different time intervals. Trastuzumab for present research was residual part from the clinical practice. The dosage ascended every 7 days (1 to 10 μg/ml). Prior studies indicated that 10 μg/ml was the saturation dose (SD) for SKBR-3 cells (Mittendorf et al., 2006). The cells keeping alive for 7 days during the dosage ascending were cultured and submitted to the subsequent treatment with SD. As the growth rate became synchronous with the parental wild type (WT-SKBR-3) cells, we finally obtained the Trastuzumab-resistant cells (TR-SKBR-3).

### Relationship Between YAP/TAZ Expression and Trastuzumab Resistance

**Figure 1:**
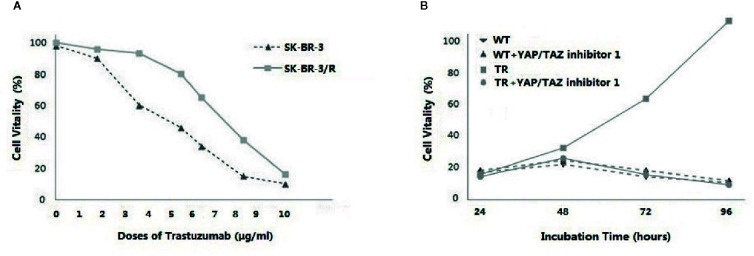
Dose-dependent response was evaluated at different concentrations of Trastuzumab (1 to 10 μg/ml), ascending every 7 days. Cells keeping alive for 7 days during the dosage ascending were collected and cultured with 10μg/ml Trastuzumab. As the growth rate became synchronous with the parental wild type (WT-SKBR-3) cells, the Trastuzumab-resistant cells (TR-SKBR-3) were obtained. Cell vitality of WT-SKBR-3 and TR-SKBR-3 was exactly similar at the starting dose. Subsequently the gap of survival rate became obvious as dose ascending **(A)**. Furthermore, YAP/TAZ inhibitor-1 (Medchemexpress LLC, New Jersy, USA) was synchronously employed in WT-SKBR-3 and TR-SKBR-3 cells while SD of Trastuzumab was performed, in order to evaluate the relationship of YAP/TAZ expression and therapeutic efficacy of Trastuzumab.YAP/TAZ inhibitor reversed the drug resistance, thereby inducing inhibition of vitality in TR cells. By contrast, TR cells without YAP/TAZ inhibitor always retained the outstanding advantage in vitality **(B)**.

As shown in **Figure 1A**, cell vitality of WT-SKBR-3 and TR-SKBR-3 was exactly similar at the starting dose. Subsequently the gap of survival rate became obvious as dose ascending. Furthermore, we synchronously employed YAP/TAZ inhibitor-1 in WT-SKBR-3 and TR-SKBR-3 cells while SD of Trastuzumab was performed. As shown in **Figure 1B**, we observed outstanding difference of vitality between TR cells and WT cells. YAP/TAZ inhibitor reversed Trastuzumab resistance in TR cells, thereby inducing obvious inhibition of their growth rate. By contrast, TR cells always retained outstanding advantage in vitality when YAP/TAZ inhibitor was not performed. These results indicated that over-expression of YAP/TAZ contributed to the resistance of tumor cells to Trastuzumab.

**Figure 2:**
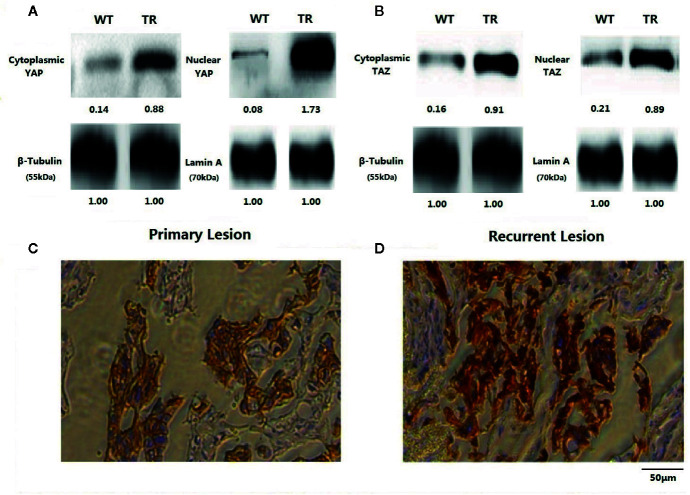
Results of western blot was shown according to the localization of YAP and TAZ. As the weight of reference protein noted, we affixed relative grey level under each band. Nuclear expression of YAP was more outstanding than in cytoplasm **(A)**. Expression of YAP and TAZ were both remarkable in TR-SKBR-3 cells **(A, B)**. Staining intensity of YAP/TAZ in recurrent lesions after first-line Trastuzumab treatment was also significantly stronger than their primary lesions **(C, D)**.

To explore the relationship between YAP/TAZ expression and Trastuzumab resistance, we further estimated YAP/TAZ expression as well as HER3 and HER2/HER3 heterodimer in TR-SKBR-3 cells and WT-SKBR-3 cells. As shown in **Figures 2A, B**, YAP/TAZ expression was generally remarkable in TR-SKBR-3 cells, and nuclear expression of YAP was more outstanding than in cytoplasm. These results correlated over-expression of YAP/TAZ with Trastuzumab resistance and indicated potential localization-dependent expression of YAP/TAZ in TR-SKBR-3 cells. Correspondingly, we also observed remarkable expression of YAP/TAZ in recurrence cases in comparison with their matching primary lesions, which supported the above preclinical findings (**Figures 2C, D**).

**Figure 3:**
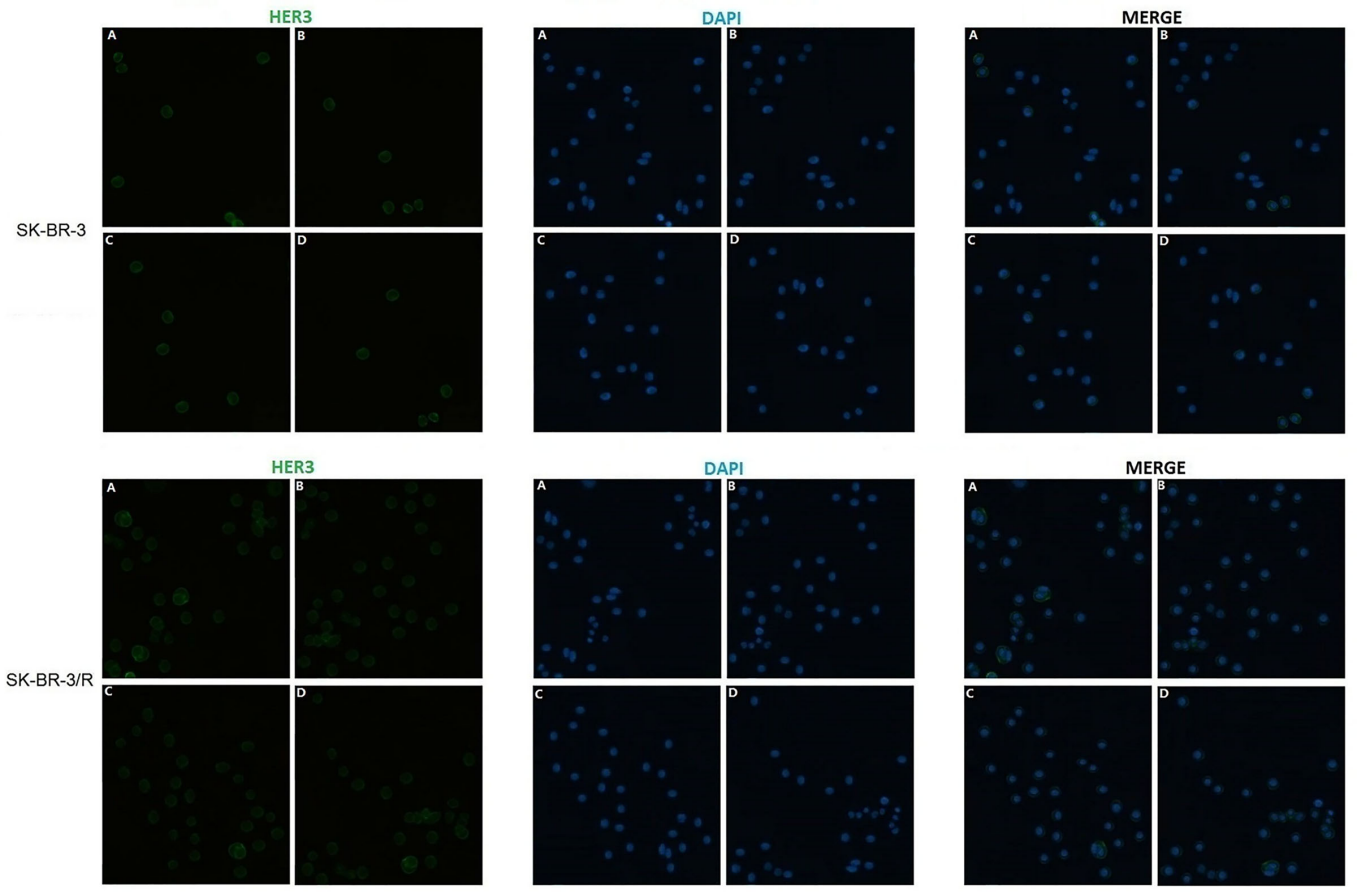
Outstanding increase of HER-3 expression was revealed in TR cells in comparison with WT cells.

**Figure 4:**
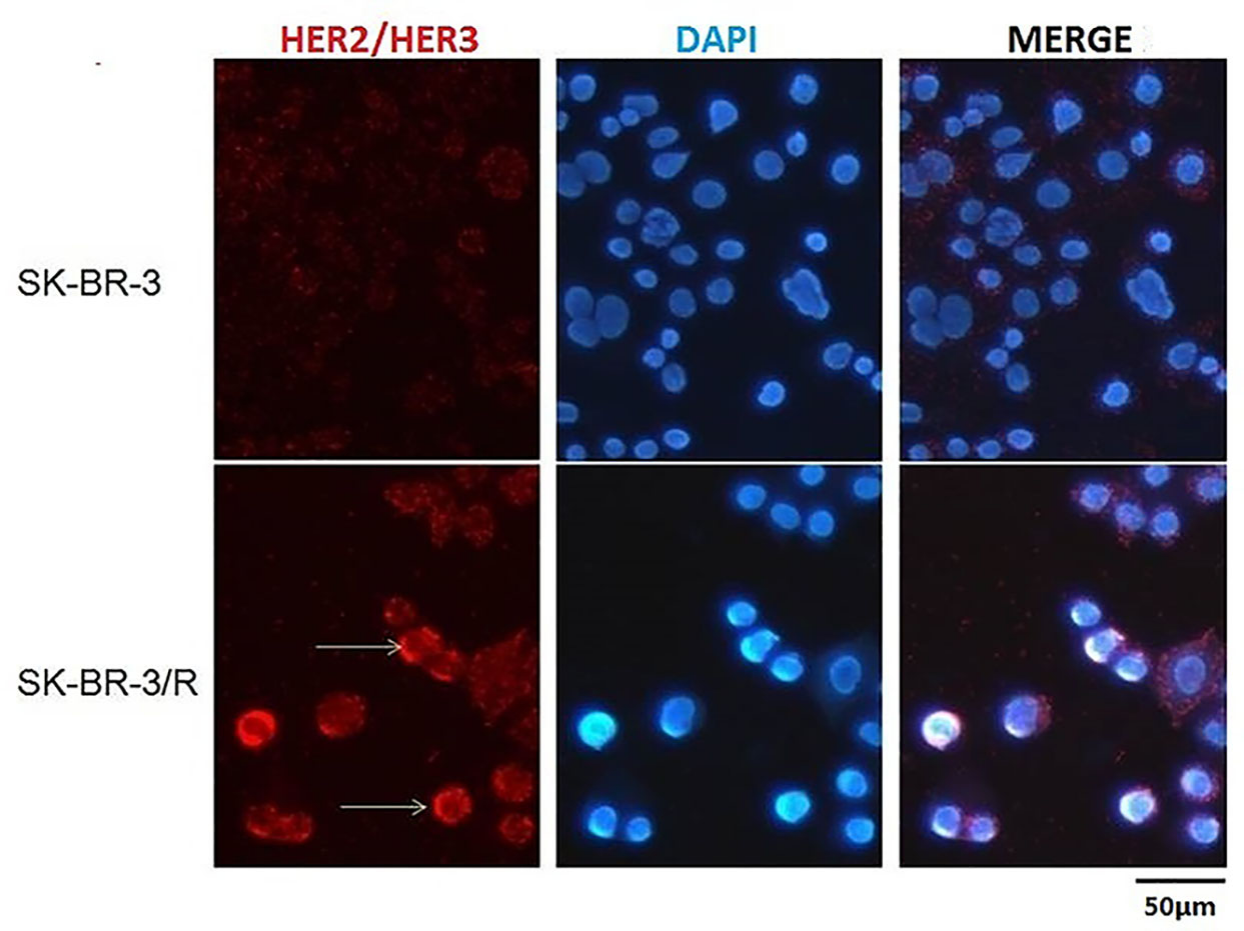
Outstanding increase of HER2/HER3 heterodimer intensity was revealed in TR cells in comparison with WT cells.

To estimate the relationship between HER2/HER3 heterodimer and Trastuzumab resistance, immunodetection assay was performed in the protein obtained from TR cells and WT cells. As shown in **Figures 3** and **4**, over-expression of HER-3 and HER2/HER3 heterodimer was obvious in TR cells in comparison with WT cells. Our findings revealed that HER-3 and HER2/HER3 heterodimer intensity was outstanding in Trastuzumab resistance cells, which were also the YAP/TAZ dominant cells. The synchronous over-expression of YAP/TAZ and HER2/HER3 heterodimer in TR cells correlated the YAP/TAZ- HER2/HER3 crosstalk with Trastuzumab resistance.

### Therapeutic Significance of YAP/TAZ Expression

We evaluated the therapeutic effect of variables (e.g., age, menopause status, cTNM stage, histological grade, Ki67, axillary lymph nodes status, RTDI, HR status, and YAP/TAZ status) and showed the results in **Table 2**. Tumor remission was outstanding in YAP/TAZ deficiency cases (p=0.035 for clinical remission and p=0.024 for pathological remission), suggesting that deficient YAP/TAZ expression contributed to better therapeutic response. Insufficient HR intensity and high chemotherapy dosage also contributed to favorable tumor remission (**Table 2**). The therapeutic effect of YAP/TAZ inferiority and HR insufficiency was outstanding when RTDI>85% (**Table 3**). Therapeutic superiority of YAP/TAZ deficiency was also amplified in HR negative patients (**Table 4**). These results indicated that contribution of YAP/TAZ expression to therapeutic response depended on chemotherapy dosage and HR status.

**Table 2:** Association between variables and therapeutic response.

Variables	No.	Clinical response		Pathological response			
		ORR (%)^1^	*P*	pCR (%)	OR	95%CI	*P*
Age							
≤50	254	213 (83.86)	0.526	65 (25.59)	0.836	0.545–1.231	0.189
>50	143	127 (88.81)		39 (27.27)			
Menopause							
Yes	188	163 (86.70)	0.379	49 (26.06)	1.192	0.522–1.340	0.483
No	209	177 (84.69)		55 (26.32)			
cTNM stage							
II	191	168 (87.96)	0.247	50 (26.18)	0.982	0.836–1.407	0.874
III	206	172 (83.50)		54 (26.21)			
Histo-grade							
I-II	216	190 (87.96)	0.883	59 (27.31)	0.985	0.679–1.218	0.260
III	181	150 (82.87)		45 (24.86)			
LN after NAT							
>3	281	238 (84.70)	0.567	74 (26.33)	1.145	0.768–1.374	0.597
≤3	116	102 (87.93)		30 (25.86)			
HR status							
HR+	240	195 (81.25)	0.028	55 (22.92)	0.742	0.508–0.933	0.045
HR-	157	145 (92.36)		49 (31.21)			
Ki67							
>14%	223	191 (85.65)	0.285	58 (26.01)	1.143	0.752–1.393	0.534
≤14%	174	149 (85.63)		46 (26.44)			
YAP/TAZ score							
≤0.5	226	209 (92.48)	0.035	75 (33.19)	0.570	0.482–0.894	0.024
>0.5	171	131 (76.61)		29 (16.96)			
RTDI							
>85%	273	250 (91.58)	0.041	78 (28.57)	0.776	0.517–0.904	0.039
≤85%	124	90 (72.58)		26 (20.97)			

^1^In this study, 340 patients were observed clinical response (complete or partial remission).

**Table 3:** Dose dependent therapeutic response of YAP/TAZ and hormone receptor (HR) status.

Variables	Therapy response							
	RTDI ≤ 85%				RTDI >85%			
	ORR (%)	*P*	pCR	*P*	ORR (%)	*P*	pCR	*P*
YAP/TAZ status								
≤0.5	79.52	0.314	10/57	0.096	98.36	0.035	65/169	0.012
>0.5	71.13		16/67		78.07		13/104	
HR status								
HR+	70.35	0.078	15/75	0.102	84.63	0.041	40/165	0.005
HR-	78.80		11/49		97.98		38/108	

**Table 4:** Dose dependent therapeutic efficacy of YAP/TAZ in different hormone receptor (HR) status.

HR status	YAP/TAZ status	Therapy response							
		RTDI ≤ 85%				RTDI >85%			
		ORR (%)	*P*	pCR	*P*	ORR (%)	*P*	pCR	*P*
Positive	≤0.5	78.47	0.691	6/35	0.154	96.34	0.047	29/99	0.038
	>0.5	66.10		9/40		80.67		11/66	
Negative	≤0.5	82.66	0.352	4/22	0.098	99.01	0.011	36/70	0.005
	>0.5	74.34		7/27		77.06		2/38	

### Prognostic Significance of YAP/TAZ Expression

**Figure 5:**
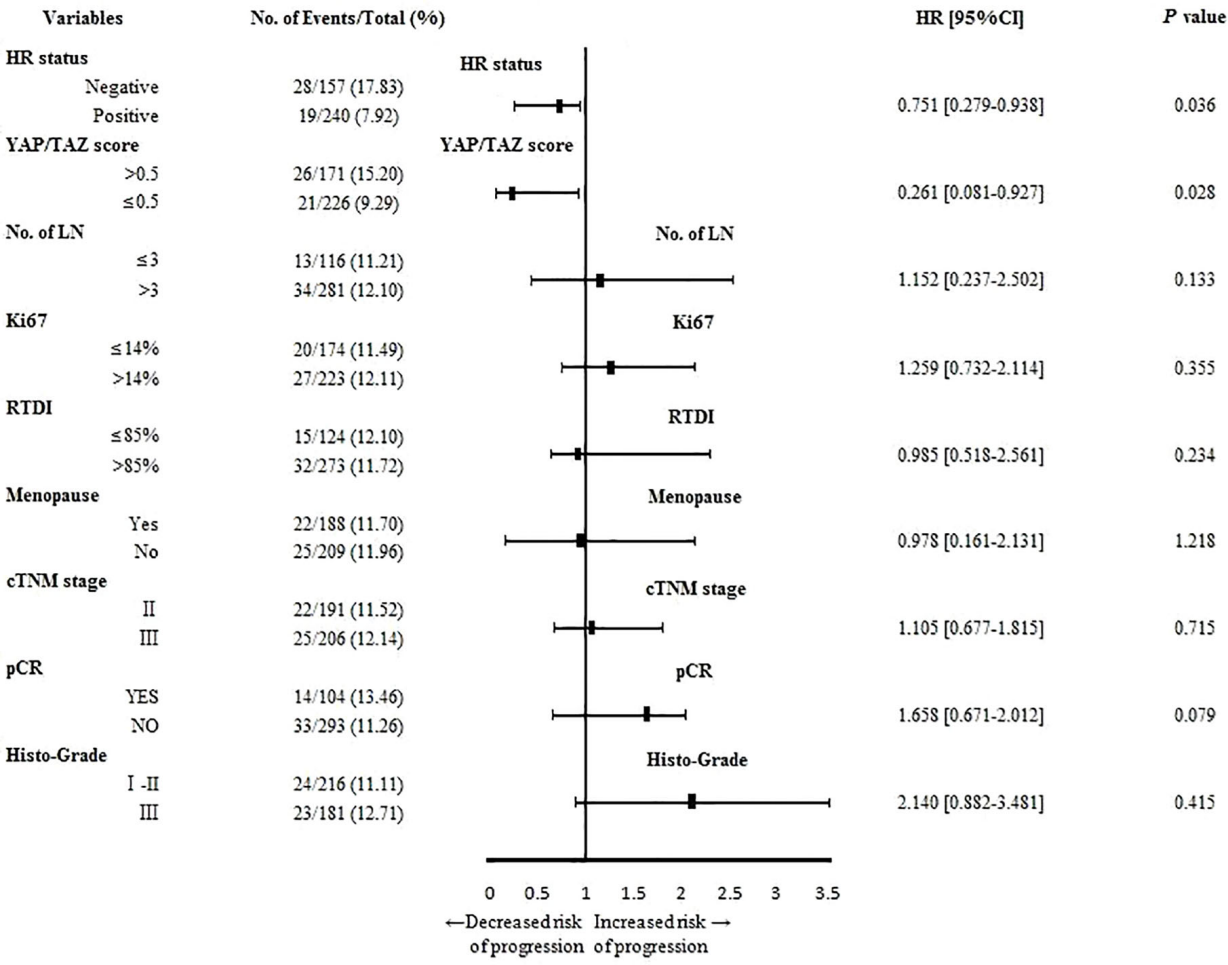
YAP/TAZ insufficiency (p=0.028, OR=0.261, 95%CI 0.081–0.927) and positive HR status (p=0.036, OR=0.751, 95%CI 0.279–0.938) contributed to reduce relapse risk of breast cancer.

**Figure 6:**
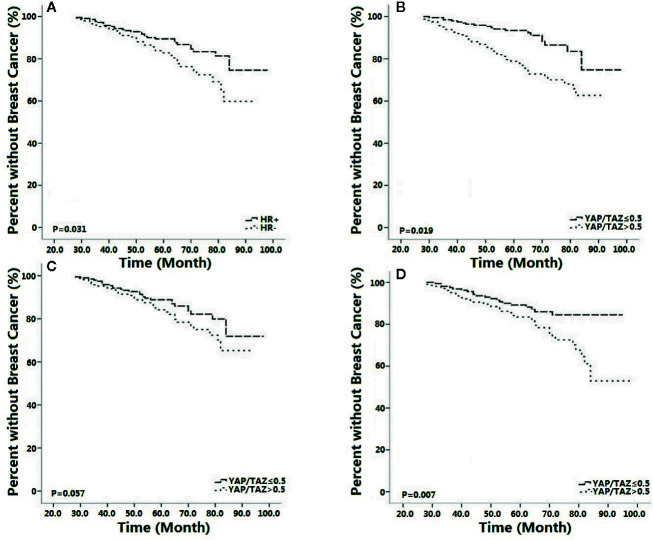
Positive hormone receptor (HR) status [(p=0.031, **(A)]** and inferior YAP/TAZ intensity [(p=0.019, **(B)**] both improved breast cancer free survival. Compared with HR positive cases [(p=0.057, **(C)**], YAP/TAZ insufficiency was more likely to improve outcomes of HR negative patients [(p=0.007, **(D)**].

As shown in **Figure 5**, inferior YAP/TAZ intensity (p=0.028, OR=0.261, 95%CI 0.081–0.927) and superior HR intensity (p=0.036, OR=0.751, 95%CI 0.279–0.938) correlated with lower recurrence risk. As shown in results of Kaplan-Meier survival analysis, superior HR intensity (p=0.031, **Figure 6A**) and inferior YAP/TAZ intensity (p=0.019, **Figure 6B**) both contributed to improvement of breast cancer-free survival. Compared with HR abundant cases (p=0.057, **Figure 6C**), deficient YAP/TAZ intensity tended to play a more important role in improving the prognosis of HR insufficient patients (p=0.007, **Figure 6D**). These results suggested mutual and interactive prognostic effect of HR intensity and YAP/TAZ expression.

### Prognostic Significance of HER3 in Residual Tumors After NAT

According to preclinical study and preliminary clinical research, expression of YAP/TAZ correlated Trastuzumab-resistance and obviously influenced prognostic outcome. In addition, we also noted significant difference of HER3 expression between TR cells and WT cells. Considering the synchronous over-expression of YAP/TAZ and HER2/HER3 heterodimer in TR cells, we hypothesized a potential relationship between HER3 expression and survival in YAP/TAZ sufficient subpopulation. We further evaluated the continuous and composite measure of recurrence risk *via* Cox model including HR, HER2, HER3, and YAP/TAZ in residual tumors, to assess their prognosis effect. Subpopulations with sufficient YAP/TAZ expression generally suffered high composite risk.

Overall, breast cancer-free survival was 90.4% (265/293), ranging from 93.6% in lowest composite risk quartile to 38.9% in highest composite risk quartile. The continuum of composite risk was also illustrated, ranging from 0.12 in lowest composite risk subpopulation to 3.24 in highest composite risk subpopulation.

**Figure 7:**
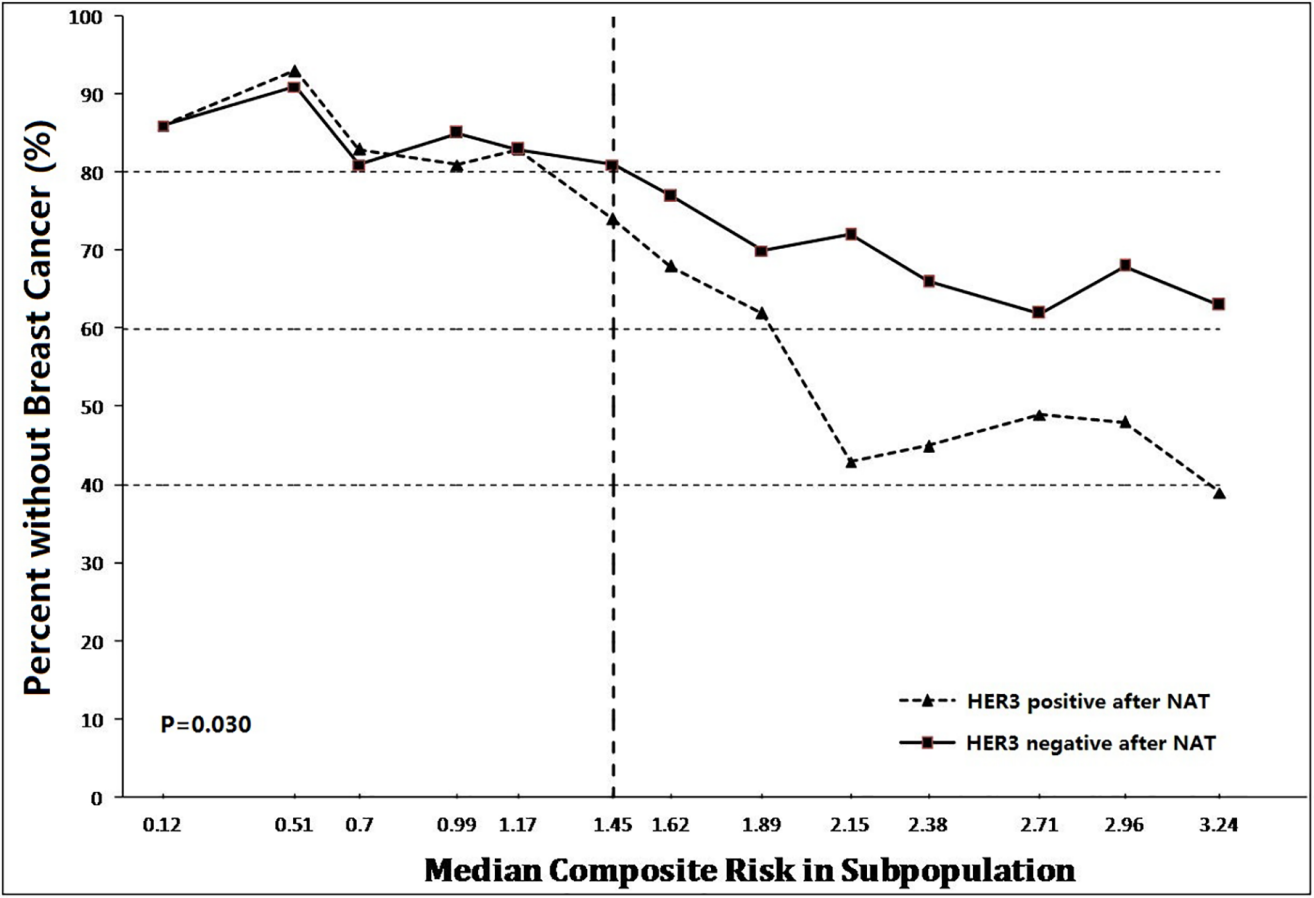
Prognostic benefit of residual HER3 negative populations was consistently significant when composite risk>1. Thereafter, the discrepancy of survival between subpopulations was absolutely raised synchronously with the continuous increasing of composite risk (p=0.03).

**Figure 8:**
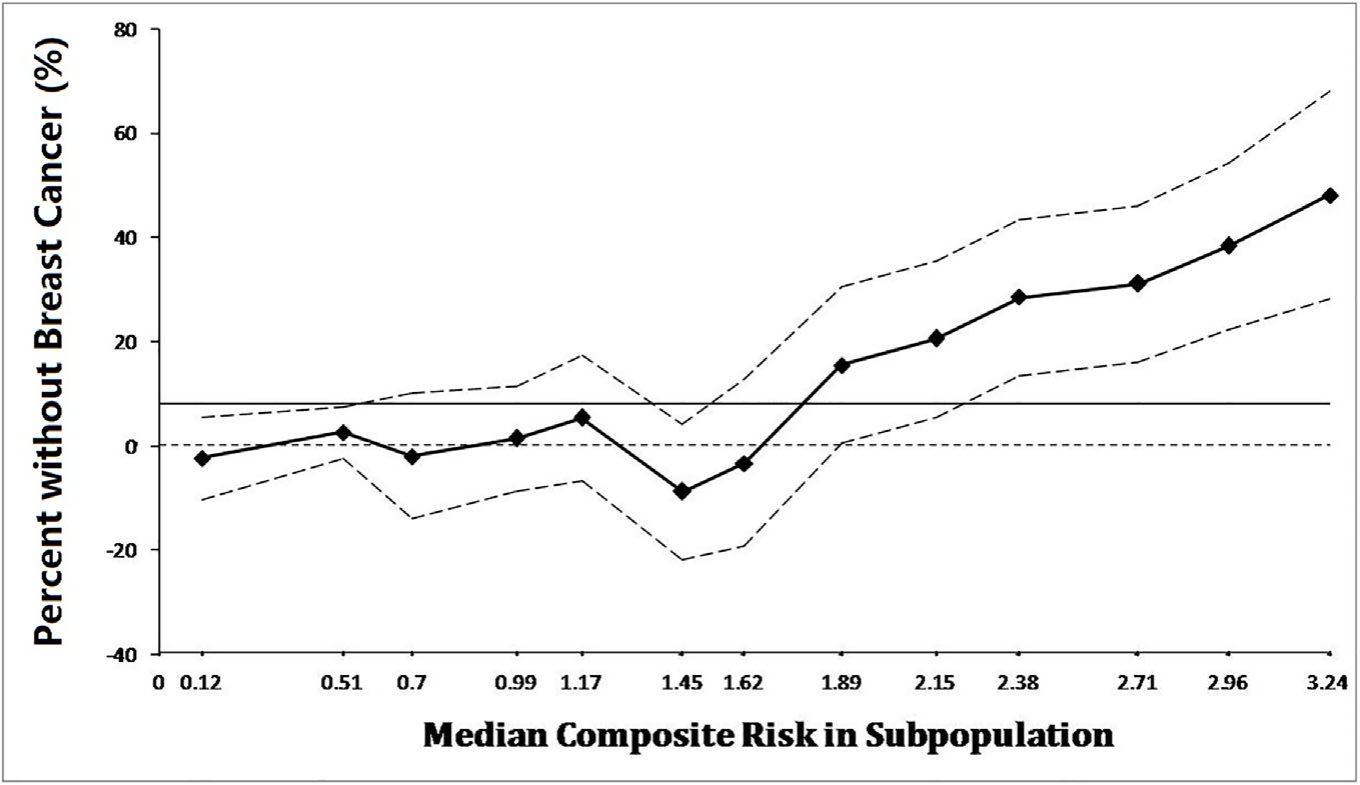
Survival of subpopulations was extremely similar when composite risk was low. In contrast, prognostic superiority of residual HER3 negative subpopulation was outstanding when composite risk rise, as their relapse free survival (>60%) was 40% more than HER3 positive cases (nearly 20%) when composite risk reached the highest point of 3.24.

As shown in **Figure 7**, prognostic benefit of residual HER3 negative populations was consistently significant when composite risk>1. The discrepancy of survival between subpopulations absolutely rose synchronously with the continuous increasing of composite risk. As shown in **Figure 8**, survival benefit of subpopulations was similar when composite risk was low. In contrast, prognostic superiority of residual HER3 negative subpopulation was outstanding when composite risk rise, as their relapse free survival (>60%) was 40% more than HER3 positive cases (nearly 20%) when composite risk reached the highest point of 3.24(p=0.03). These findings revealed that cases with synchronous over-expression of YAP/TAZ and HER3 tended to suffer poor survival, which generally accorded with the results of the above *in vitro* experiments.

## Discussion

YAP and TAZ were transcriptional co-activators ubiquitously related with tissue development, and involved in the invasion of breast cancer (Bartucci et al., 2015). They obtained phosphorylation through the Hippo pathway and gained activation *via* cellular density (Zhao et al., 2007; Ota and Sasaki, 2008; Kim et al., 2011). TAZ played important role in the occurrence of breast cancer drug resistance (Cordenonsi et al., 2011). In this study, we revealed the synchronous over-expression of YAP/TAZ as well as HER-3 and HER2/HER3 heterodimer in Trastuzumab-resistant cell lines. We found that inhibition of YAP/TAZ expression reversed Trastuzumab resistance. We clarified the outstanding effect of YAP/TAZ expression in therapeutic response and survival of HER2 positive patients. We also revealed that synchronous over-expression of YAP/TAZ and HER3 contributed to poor survival, which supported the potential prognostic effect of YAP/TAZ-HER2/HER3 crosstalk.

### Relationship Between YAP/TAZ and Multiple Cancer-Associated Features

YAP/TAZ expression widely involved in migration and invasion of breast cancer cells (Mi et al., 2015). Knockdown of YAP/TAZ reduced the above migration and invasion (Chan et al., 2008). TAZ promoted a luminal to basal lineage switch, which was confirmed by its depletion in basal and epithelial cells promoted luminal differentiation (Skibinski et al., 2014). Prior studies also reported the similar transforming potential of YAP. Over-expression of YAP caused inhibition of apoptosis and anchorage-independent growth, which induced tumorigenic transformation (Overholtzer et al., 2006). Inhibition of YAP suppressed tumor development and tumor metastasis in a mouse model of breast cancer, suggested that cooperating genetic events were necessary for generating a neoplastic phenotype (Chen et al., 2014). At the preclinical research level, prior literatures prompted that YAP/TAZ played an important role in resistance to anti-cancer drugs and other cancer-associated features such as tumor cell migration and metastasis. At clinical study level, YAP/TAZ expression also related to tumor metastasis and prognostic outcomes.

### Role of YAP/TAZ in Breast Cancer Outcomes

Hippo pathway was essential in various pathological processes of breast cancer development. Disturbance Hippo pathway promoted breast cancer metastasis through multiple mechanisms. As the crucial component of Hippo pathway, YAP/TAZ expression played a critical role in tumor cell migration and colonization in tissues (Bos et al., 2009). Prior studies evaluated relationship between YAP/TAZ expression and survival of breast cancer patients, and explored the potential function of YAP/TAZ as a predictive clinical biomarker.

Prior studies had proved that over-expression of YAP related to tumorigenicity (Wang et al., 2012). YAP dysfunction relieved lung metastasis in a genetically engineered mouse model of breast cancer. Phosphorylated HER3 caused activation of YAP/TAZ in tumor cells, which finally induced bone metastasis (Li et al., 2017). Nuclear expression of TAZ in bone metastasis lesion was significantly higher than its expression in primary tumors (Bartucci et al., 2015).

Mutation in the Hippo signaling pathway also contributed to chemo-resistance of cancer cells, while the absence of TAZ obviously defused the chemo-resistance (Bartucci et al., 2015). Prior studies indicated that over-expression of TAZ promoted chemo-resistance in MCF10 breast cell line and depressed the chemo-sensitivity of MDA-MB-231 breast cell line (Lai et al., 2011). Cultured MCF-10A cell line was competent for YAP activation in invasive breast cancer (Lee et al., 2019). The MDA-MB-231 breast cancer cell line and MCF-7 cell lines were both qualified in research of YAP related breast cancer progression (Hua et al., 2015). Prior study based on MDA-MB-468 and human breast cancer cell line ZR-75-30 had also indicated that YAP/TAZ promoted breast cancer metastasis (Wang et al., 2018).

Patients with superior TAZ expression suffered high risk of tumor relapse and poor outcomes. Over-expression of TAZ caused obvious decline of recurrence-free survival (51.7% in over-expression group versus 78% in negative group; p=0.014) (Bartucci et al., 2015). Activation of TAZ during the metastatic procedure was also observed by comparing primary and metastases lesions (Matteucci et al., 2013). Staining intensity and cellular localization of TAZ brought out a TAZ-based score, which predicted the pathological response of NAT-treated HER2 positive breast cancer (Vici et al., 2014). Over-expression of TAZ might induce residues of HER2-positive tumors after NAT, and high nuclear intensity of TAZ induced poor clinical outcomes (Di Benedetto et al., 2017).

As shown in this study, tumor remission was significant in patients with inferior YAP/TAZ intensity, suggesting that YAP/TAZ expression might relate to the therapeutic efficacy of NAT. Tumor remission of YAP/TAZ insufficient cases was outstanding when RTDI>85%, suggested that therapeutic superiority of insufficient YAP/TAZ was dose-depended (**Tables 2**–**4**). Inferior YAP/TAZ intensity also contributed to reduce the risk of relapse and induce encouraging survival (**Figures 5** and **6**), and these results were similar with the previous findings. According to the results of *in vitro* experiments, over-expression of YAP/TAZ was obvious in Trastuzumab-resistance cells (**Figure 2**), suggested that superior intensity of YAP/TAZ might contribute to the occurrence of drug resistance. Inhibition of YAP/TAZ reversed the above resistance, thereby resuming the therapeutic efficacy of Trastuzumab (**Figure 1**). According to the clinical study of this program, insufficient expression of YAP/TAZ contributed to better survival, while over-expression of YAP/TAZ raised the relapse risk. Correspondingly, we indeed observed remarkable expression of YAP/TAZ in recurrence lesions. These encouraging preclinical and clinical findings provided ideas in the treatment of Trastuzumab resistant cases. Moreover, with the further research of Trastuzumab resistance, doctors should pay attention to individualized treatment. We needed adjusted therapy according to drug sensitivity, without monotony or repetitious tasks. Early intervention might reduce disease relapse in potential drug resistance cases.

### Different Trait of YAP/TAZ According to Distinct Subtypes

Prior studies clarified that YAP/TAZ affected the biological behavior of tumor cells according to molecular subtypes of breast cancer (Diaz-Martin et al., 2015). Analysis of TAZ expression in 640 distinct phenotypes of breast cancer patients suggested that over-expression of TAZ was obviously associated with negative HR status, while other literatures reported over-expression of TAZ in HR positive breast cancer (Bartucci et al., 2015; Wang et al., 2016).

Over-expression of YAP/TAZ appeared to be a shared trait according to the intrinsic subtypes, which was associated with prognostic outcomes (Vici et al., 2014; Skibinski et al., 2014; Kim et al., 2014; Bartucci et al., 2015). Superior expression of TAZ was confirmed in basal-like cases in comparison with HR positive patients (Skibinski et al., 2014). Over-expression of TAZ also caused the descent of survival in basal-like cases (Skibinski et al., 2014), and tended to appear synchronously with the existence of HER2 positive subtype (Bartucci et al., 2015). Preclinical studies also claimed that over-expression TAZ was present in HER2-driven mammary tumors (Serrano et al., 2013).

As shown in this study, negative status of HR contributed to tumor remission, and therapeutic superiority of inferior YAP/TAZ intensity was upward in HR negative patients (**Tables 2**–**4**). Although inferior intensity of YAP/TAZ and positive HR status contributed to reduce the risk of relapse and improve survival, superiority of YAP/TAZ insufficiency was more likely to be amplified in HR negative cases (**Figures 5** and **6**). These results suggested that contributions of YAP/TAZ expression to therapeutic efficacy and prognostic outcomes obviously depended on HR status. According to our findings, therapeutic and prognostic effect of HR and YAP/TAZ was mutual and interactive. Inferior HR intensity amplified the therapeutic and prognostic advantage of YAP/TAZ insufficient cases.

### Crosstalk Between Hippo Pathway and Other Signaling Pathways

Wide crosstalk between Hippo pathway and other signaling pathways formed complex cellular signaling networks, which obviously affected the development and metastasis of tumors. The activation of AKT increased the probability of YAP to boost the proliferation of MCF10A cells (Overholtzer et al., 2006). Knockdown of YAP inhibited a series of cytokines and vascular invasion of breast cancer cells (Sharif et al., 2015). These prior studies suggested that breast cancer cells might regulate vascular invasiveness *via* YAP and Hippo pathway.

YAP directly activated Pik3cb expression, while YAP required Pik3cb to promote cells proliferation and activate the AKT pathway (Lin et al., 2015). Pik3cb served as a crucial association between Hippo-YAP and PI3K-AKT pathways (Lin et al., 2015). Mutually stimulatory cross-talk between YAP and PI3K established a feed forward regulatory circuit. YAP increased expression of the PI3K subunit Pik3cb, and PI3K stimulated YAP activity, thereby promoting tumor cells proliferation and survival (Lin et al., 2015). Prior studies also reported that YAP regulated cell metabolism, which was the well-described function of PI3K-AKT signaling (Lin et al., 2015).

ERβ obviously influenced the activation of HER2/HER3/Akt pathways, while the activated HER2/HER3 heterodimer indicated notable activation of PI3K/Akt pathway (Lindberg et al., 2011). Existing of ERβ obviously inhibited the phosphorylated procedure of HER2/HER3 (Lindberg et al., 2011). Previous literatures also observed the up-regulation of HER2 and down-regulation of HER3 in ERβ over-expression cells (Lindberg et al., 2011).

Based on these findings, we attempted to explore the relationship between YAP/TAZ expression and Trastuzumab resistance. As we observed in our preclinical study, HER-3 and HER2/HER3 heterodimer intensity was outstanding in Trastuzumab resistance cells (**Figures 3** and **4**), which was also the YAP/TAZ dominant cells. The synchronous over-expression of YAP/TAZ and HER2/HER3 heterodimer suggested the crosstalk between Hippo-YAP and PI3K-AKT signaling pathways. According to the activation effect of ERβ to HER2/HER3 and Akt pathways, the above crosstalk was more obvious in ER insufficiency cases. Correspondingly, we observed outstanding prognostic inferiority in cases who suffered synchronous over-expression of YAP/TAZ and HER3 (**Figures 7** and **8**). Considering the similar synchronous over-expression in Trastuzumab resistance cells, we believed that YAP/TAZ- HER2/HER3 crosstalk played crucial role in prognosis of HER2 positive patients.

As a single center retrospective study, we were aware the following limitations of our results. Considering the potential localization-dependent expression, we should focus on the nuclear intensity of YAP/TAZ in our future work. Further study should pay more attention to the comparison of primary and residual lesions, which might carry out more meaningful ideas to research about Trastuzumab resistance. Both the primary and recurrence expression of YAP/TAZ should be considered in calculation of composite risk. Besides the assessment of YAP/TAZ in the tumor cells, further studies should also focus on the stromal tissues, thereby identifying the appropriate micro-environment for YAP/TAZ activation. As the above improvement carried out, YAP/TAZ-based biomarker would be more effective for the therapeutic and prognostic evaluation.

## Conclusions

Over-expression of YAP/TAZ as well as HER-3 and HER2/HER3 heterodimer was synchronously remarkable in Trastuzumab-resistant cell lines. Inhibition of YAP/TAZ expression reversed Trastuzumab resistance. Deficient YAP/TAZ intensity as well as insufficient hormone receptor intensity and high chemotherapy dosage contributed to favorable therapeutic response. Deficient YAP/TAZ intensity and abundant hormone receptor intensity contributed to better survival, and so did absence of HER3 expression in residual lesions. Prognostic superiority of YAP/TAZ expression depended on hormone receptor status. Cases with synchronous over-expression of YAP/TAZ and HER3 suffered poor survival, which revealed the potential effect of YAP/TAZ-HER2/HER3 crosstalk in prognosis of HER2-positive patients.

## Data Availability Statement

All datasets generated for this study are included in the article/supplementary material.

## Ethics Statement

The studies involving human participants were reviewed and approved by Xiangya Hospital Ethics Committee. The patients/participants provided their written informed consent to participate in this study.

## Author Contributions

J-QY and ZX: concept, design, definition of intellectual content, manuscript review. JY: literature search, clinical studies, experimental studies. JY and N-HD: data acquisition, data analysis, statistical analysis, manuscript preparation, manuscript editing. All authors contributed to the article and approved the submitted version.

## Conflict of Interest

The authors declare that the research was conducted in the absence of any commercial or financial relationships that could be construed as a potential conflict of interest.
